# Receptor binding specificity of recent human H3N2 influenza viruses

**DOI:** 10.1186/1743-422X-4-42

**Published:** 2007-05-09

**Authors:** Kshama Kumari, Shelly Gulati, David F Smith, Upma Gulati, Richard D Cummings, Gillian M Air

**Affiliations:** 1 Department of Biochemistry & Molecular Biology, University of Oklahoma Health Sciences Center, Oklahoma City, OK, USA; 2 Department of Biochemistry and Consortium for Functional Glycomics Core H, Emory University School of Medicine, Atlanta GA, USA

## Abstract

**Background:**

Human influenza viruses are known to bind to sialic acid linked α2-6 to galactose, but the binding specificity beyond that linkage has not been systematically examined. H3N2 human influenza isolates lost binding to chicken red cells in the 1990s but viruses isolated since 2003 have re-acquired the ability to agglutinate chicken erythrocytes. We have investigated specificity of binding, changes in hemagglutinin sequence of the recent viruses and the role of sialic acid in productive infection.

**Results:**

Viruses that agglutinate, or do not agglutinate, chicken red cells show identical binding to a Glycan Array of 264 oligosaccharides, binding exclusively to a subset of α2-6-sialylsaccharides. We identified an amino acid change in hemagglutinin that seemed to correlate with chicken red cell binding but when tested by mutagenesis there was no effect. Recombinant hemagglutinins expressed on Sf-9 cells bound chicken red cells but the released recombinant baculoviruses agglutinated only human red cells. Similarly, an isolate that does not agglutinate chicken red cells show hemadsorption of chicken red cells to infected MDCK cells. We suggest that binding of chicken red cells to cell surface hemagglutinin but not to virions is due to a more favorable hemagglutinin density on the cell surface. We investigated whether a virus specific for α2-6 sialyloligosaccharides shows differential entry into cells that have varying proportions of α2-6 and α2-3 sialic acids, including human A549 and HeLa cells with high levels of α2-6 sialic acid, and CHO cells that have only α2-3 sialic acid. We found that the virus enters all cell types tested and synthesizes viral nucleoprotein, localized in the nucleus, and hemagglutinin, transported to the cell surface, but infectious progeny viruses were released only from MDCK cells.

**Conclusion:**

Agglutination of chicken red cells does not correlate with altered binding to any oligosaccharide on the Glycan Array, and may result from increased avidity due to density of hemagglutinin and not increased affinity. Absence of α2-6 sialic acid does not protect a cell from influenza infection and the presence of high levels of α2-6-sialic acids on a cell surface does not guarantee productive replication of a virus with α2-6 receptor specificity.

## Background

Influenza hemagglutinin binds to sialic acids attached to membrane glycosphingolipids, proteoglycans, and glycoproteins [1]. It was shown that the linkage between sialic acid and the second sugar (usually galactose) could determine whether an influenza virus binds to avian or human cells [2,3], and that a single mutation in the hemagglutinin (HA) was sufficient to change binding from α2-3 to α2-6 and host cells from avian to human, or vice versa [4,5]. Now it is generally accepted that human influenza viruses bind to α2-6 sialic acids and avian viruses bind to α2-3 sialic acids [6-8], while swine influenza viruses are reported to bind mainly α2-6 sialic acids but also α2-3 sialic acid receptors [9,10]. We previously found that influenza viruses with HA from A/NWS/33 can bind to α2-8 linked sialic acids [11], raising the possibility of other forms of sialic acid receptor in human influenza infection. A solid phase assay with multivalent sialylsaccharides attached to a polyacrylamide backbone showed that human isolates from the beginning of the H2N2 pandemic in 1957 and the 1968 H3N2 pandemic had four-fold higher binding affinity to 6'sialyllactosamine than avian viruses [12]. It was suggested that a change in receptor binding specificity is a prerequisite for effective transmission and replication to cause a new human pandemic. However, some early H2 viruses preferentially bind 3'sialyllactose [2,13] and the H5N1 isolates from humans have been shown to have avian-like (α2-3) receptor specificity [14,15], although approximately equal binding to α2-3 and α2-6 receptors was seen with two human H5N1 isolates [16].

Receptor binding specificities of recent H3N2 viruses have not been studied in detail. Human influenza viruses were able to agglutinate both human and chicken red blood cells until the early to mid 1990s, when H3N2 and H1N1 influenza viruses lost the ability to agglutinate chicken red blood cells but were still able to agglutinate human red blood cells [17-20]. The responsible mutations in H3 HA were suggested to be Glu190Asp [19] or the successive changes of Gln226-to-Leu-to-Ile-to-Val [18]. Receptor specificity of influenza viruses has been seen to correlate with ability to agglutinate erythrocytes from different species [21], although only a limited number of viruses and species of red cells were investigated.

Due to the difficulties of obtaining and working with oligosaccharides, many receptor specificity studies have compared the binding of viruses to simple trisaccharides such as α2-3- or α2-6- sialyllactosamine or sialyllactose [22,23] in a competition assay to virus bound to a plate. Alternative methods include virus overlays to gangliosides separated by thin-layer chromatography [24,25] or hemagglutination assays using red cells that were treated with sialidase then re-sialylated using specific sialyltransferases [3,26,27]. The binding constant of a single sialylated sugar chain to HA is typically in the mM range, so multivalent binding is necessary to obtain a binding signal in a solid phase format [11,28,29]. Gambaryan et al. studied the binding of duck viruses to multivalent sialyl-oligosaccharides-PAA by competing the binding with bovine fetuin labeled with horseradish peroxidase and showed that binding affinity is higher with β1-3 rather than β1-4 between the 2^nd ^and 3^rd ^sugar. Human viruses showed higher binding affinity to sialyloligosaccharides having a sulfo- group at position 6 of β1-4 linked GlcNAc [30]. The development of printed covalent glycan arrays by the Consortium for Functional Glycomics allows for the first time a clear differentiation of what sugar structures bind to a given lectin [31-33]. The array has been used to characterize the receptors for expressed HA of 1918 influenza viruses and H5N1 isolates, using multiple antibody cross-linking to achieve the necessary level of multivalency [15,34].

In the present study we used fluorescently labeled whole virions to identify potential receptors for recent human H3N2 viruses using glycan arrays. The results reveal that all the recent H3N2 viruses bind to the same selection of α2-6-sialylated oligosaccharides, and the chicken red cell agglutination activity of recent viruses is not due to acquisition of binding to α2-3 sialylsaccharides. Also we demonstrate that the presence of α2-6 sialic acid on a cell surface has little to do with its ability to be productively infected by human influenza viruses.

## Results

### Chicken red blood cell agglutination

**Figure 1:**
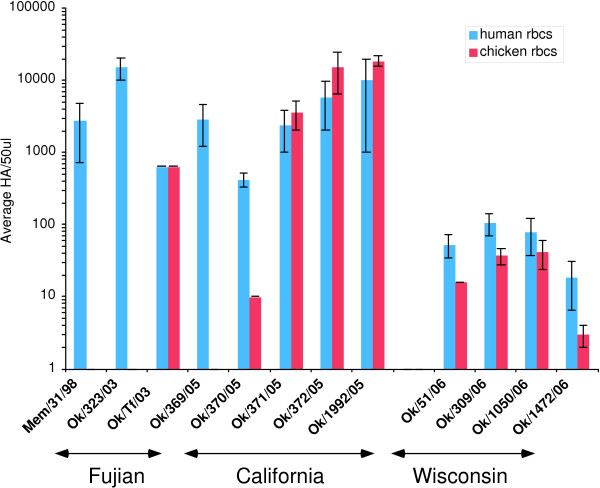
**Hemagglutination titers of recent viruses with chicken and human red blood cells**. The viruses were grown in MDCK cells and concentrated by centrifugation to obtain a ratio of HA titer with chicken red cells compared to human red cells. For Mem/31/98, OK/323/03 and OK/369/05 there was still no agglutination of chicken red blood cells. The 2006 viruses were not concentrated. The data are average ± standard deviation of 3 experiments.

Most of the recent H3N2 isolates from Oklahoma agglutinate chicken red blood cells, in marked contrast to viruses isolated from about 1996 to 2003. One out of two 2003 isolates, four out of five from 2005, and four out of four 2006 isolates agglutinated chicken cells (Fig. 1). The human to chicken cell hemagglutination ratios were close to 1:1 for OK/Tf/03, OK/371/05, OK/372/05 and OK/1992/05, and about 50:1 for OK/370/05. Memphis/98 (HG), OK/323/03, and OK/369/05 did not show any agglutination with chicken red cells at the highest concentration available (human:chicken >3000).

### Binding of fluorescently labeled viruses to glycans

**Figure 2:**
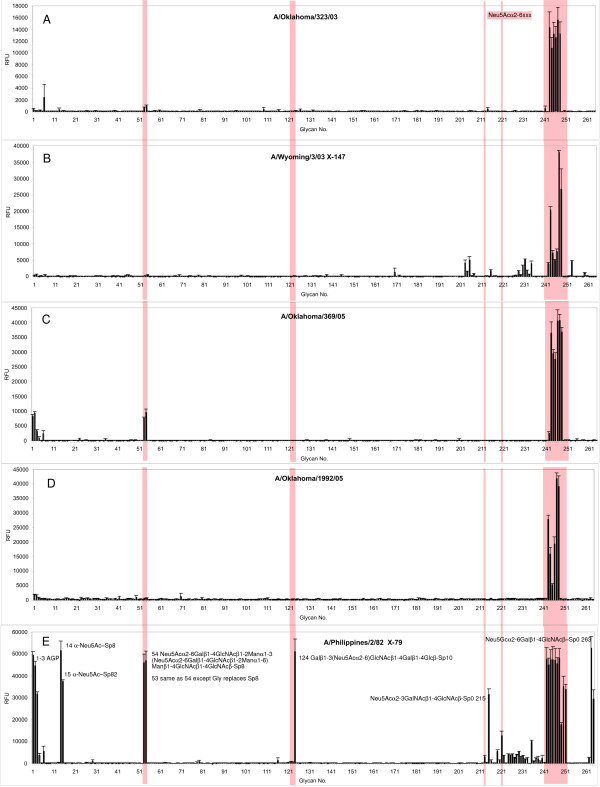
**Binding of viruses to the Glycan Array**. Glycan printed array V2 binding analyses of A/Oklahoma/323/03 (A), egg adapted vaccine reassortant A/Wyoming/03/03 X-147 (B), A/Oklahoma/369/05 (C), A/Oklahoma/1992/05 (D), and an older vaccine strain A/Phillipines/2/82 X-79 (E). The microarray slides used for these experiments had 246 glycans, shown along the X axis. The shaded regions show the α2-6-sialylsaccharides. The fluorescence is the average of 4 replicate spots ± standard error after the highest and lowest readings of six were excluded. OK/369/05 and OK/323/03, that do not bind chicken red cells, bind to the same α2-6-sialylsaccharides as do agglutinating and vaccine strain egg grown viruses.

To investigate if there is a change in receptor specificity between chicken red cell agglutinating and non-agglutinating viruses, we used the Glycan Array of Core H of the Consortium for Functional Glycomics. The printed array v2 contained 264 natural and synthetic glycans [35]. Of the 264 glycans, 76 possess sialic acid; 54 with α2-3 linkages, 22 with α2-6 linkages, and 7 with α2,8 linkages [31]. Binding was done at 4°C where the viral neuraminidase is inactive. Experiments at higher temperature designed to demonstrate cleavage of receptors by NA were not successful, so there was no need to include an NA inhibitor in the binding experiment. The array results are shown in Fig 2, and Tables 1 and 2 list the glycans bound as percent of total binding. "Total binding" is the sum of fluorescent signals of all glycans that show ≥ 5% of the highest binding species.

**Table 1:** Glycans bound by 2003–2005 H3N2 viruses

**Glycan #**	**Glycan**	Percent of total binding					
		369/05	1992/05	370/05	323/03	Wyoming	Philippines

247	Neu5Acα2-6Galβ1-4GlcNAcβ1-3Galβ1-4(Fucα1-3)GlcNAcβ1-3Galβ1-4(Fucα1-3)GlcNAcβ-Sp0	16	27	20	19	31	6
246	Neu5Acα2-6Galβ1-4GlcNAcβ-Sp8	16	13	14	16	6	6
248	Neu5Acα2-6Galβ1-4GlcNAcβ1-3Galβ1-4 GlcNAcβ-Sp0	15	26	17	16	22	6
243	Neu5Acα2-6GalNAcβ1-4GlcNAcβ-Sp0	15	18	14	18	17	6
244	Neu5Acα2-6Galβ1-4 [6OSO_3_]GlcNAcβ-Sp8	12	10	13	13	6	6
245	Neu5Acα2-6Galβ1-4GlcNAcβ-Sp0	11	3	14	16	4	6
54	Neu5Acα2-6Galβ1-4GlcNAcβ1-2Manα1-3 (Neu5Acα2-6Galβ1-4GlcNAcβ1-2Manα1-6)Manβ1-4GlcNAcβ1-4GlcNAc-Sp8	4	0	4	1	0	6
53	Neu5Acα2-6Galβ1-4GlcNAcβ1-2Manα1-3 (Neu5Acα2-6Galβ1-4GlcNAcβ1-2Manα1-6) Manβ1-4GlcNAcβ1-4GlcNAc-Gly	3	0	4	1	0	6
242	Neu5Acα2-6GalNAcα-Sp8	1	0	0	0	3	6
203	NeuAcα2-8NeuAcα2-8NeuAcα2-8NeuAcα2-3(GalNAcβ1-4)Galβ1-4Glcβ-Sp0					3	
231	Neu5Acα2-3Galβ1-4(Fucα1-3)GlcNAcβ-Sp8					4	
253	Neu5Acα2-8Neu5Acα2-3Galβ1-4Glcβ-Sp0					4	

**Table 2:** Additional glycans that bound only to Philippines/2/82 X-79^1^

Glycan #	Glycan	Philippines
263	Neu5Gcα2-6Galβ1-4GlcNAcβ-Sp0	7
14	Neu5Ac-Sp8	7
124	Galβ1-3(Neu5Acα2-6)GlcNAcβ1-4Galβ1-4Glcβ-Sp10	6
15	Neu5Acα1-2-Sp82	5
250	Neu5Acα2-6Galβ1-4Glcβ-Sp8	4
251	Neu5Acα2-6Galβ-Sp8	4
215	Neu5Acα2-3GalNAcβ 1-4GlcNAcβ-Sp0	4
264	Neu5Gcα-Sp8	4
249	Neu5Acα2-6Galβ1-4Glcβ-Sp0	2
221	Neu5Acα2-3Galβ1-3(Neu5Acα2-6)GalNAcα-Sp8	2
235	Neu5Acα2-3Galβ1-4GlcNAcβ1-3Galβ1-4GlcNAcβ1-3Galβ1-4GlcNAcβ-Sp0	1
219	Neu5Acα2-3Galβ1-3(Neu5Acα2-3Galβ1-4)GlcNAcβ-Sp8	1
229	Neu5Acα2-3Galβ1-4(Fucα1-3)GlcNAcβ1-3Galβ1-4(Fucα1-3)GlcNAcβ1-3Galβ1-4(Fucα1-3)GlcNAcβ-Sp0	1

^1 ^Results are given as percent of total binding

**Figure 3:**
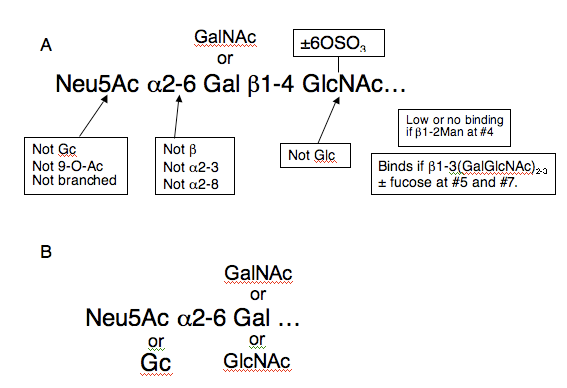
**Binding motifs of H3N2 viruses**. The minimal motifs bound by 2003–2005 H3N2 viruses (A) and A/Philippines/2/82/X-79 (B). Recent viruses require a minimum of 3 sugars to bind while Philippines binds a disaccharide. It can accommodate an N-glycolyl in place of N-acetyl on the terminal sialic acid.

All the recent H3N2 viruses, even the egg-adapted vaccine reassortant Wyoming/03 X-147, show essentially identical binding patterns. They bind to only a subset of the oligosaccharides that have α2-6 sialic acid. The overall binding motif for the 2003–2005 viruses is shown in Fig 3A. These viruses bind Neu5Acα2-6Galβ1-4GlcNAc motifs. They do not bind if N-acetylneuraminic acid is hydroxylated to the glycolyl form (Neu5Gc) or to 9-OAc sialic acid; or if there is an additional α 2,8 linked sialic acid. The GlcNAc at the third position is necessary for binding and Glc is not tolerated at this position.

The 1982 vaccine virus A/Philippines/82 X-79 has broader binding specificity as shown in Table 2 and Fig 3B. It binds to Neu5Gcα2-6 as shown by Anders 1986 [36]. Unlike the later viruses, it binds a branched NeuAcα2-6 structure and also biantennary structures branched through mannose. There is significant binding to Neu5Acα2-3GalNAcβ1-4GlcNAcβ-Sp0 but not to other α2-3 sialylated structures.

### Mutation and red cell binding assay

**Figure 4:**
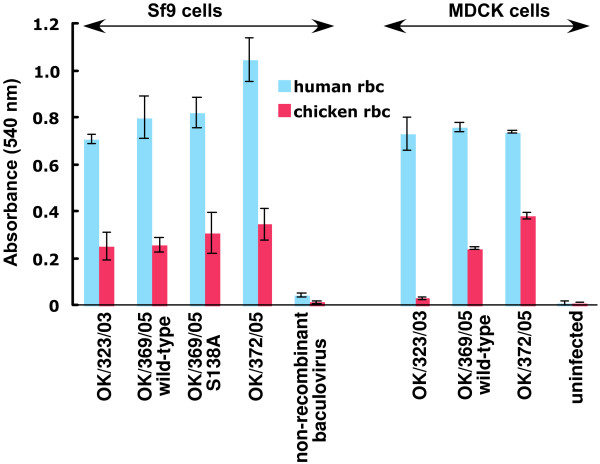
**Hemadsorption of chicken and human red blood cells to cell surface-expressed HA**. Red cells were bound to recombinant HA expressed on the surface of Sf-9 cells and to influenza HA expressed on MDCK cells. Bound red cells were lysed with water and released hemoglobin was measured at 540 nm. The results shown are an average of 4 experiments ± standard deviation.

The Glycoarray showed no specificity differences between viruses that bind chicken red cells and those that do not, suggesting the difference either involves structures that are not on the array, or it is in affinity/avidity. Sequence comparisons of HA of H3N2 viruses showed only one correlation between sequence and ability to agglutinate chicken red cells; the non-agglutinating or very low agglutinating 2005 viruses have Ser or Thr at position 138 of HA1 instead of Ala. To investigate if this substitution affects binding to chicken red cells we cloned and expressed the HA genes of OK/323/03, OK/372/05, OK/369/05 wild type and OK/369-S138A mutant in the baculovirus system. Hemadsorption to HA-expressing Sf9 cells was seen with human red cells for all constructs. Surprisingly, all the HAs on Sf9 cells bound chicken red cells, even the OK/369/05 wt and OK/323/03 HAs that did not agglutinate chicken cells in the hemagglutination assay (Fig 4). We also tested the released baculoviruses for agglutination ability. All the recombinant baculoviruses showed good HA titers with human red cells, but in contrast to the hemadsorption result on Sf9 cells, none of the released recombinant baculoviruses expressing the HAs of OK/323/03, OK/372/05, OK/369/05 wild-type and OK/369-S138A mutant were able to agglutinate chicken cells (Table 3). The hemadsorption of chicken red cells to OK/369/05 HA suggests that HA expressed in insect cells binds with higher avidity than the HAs on recombinant baculovirus or influenza virus particles.

**Table 3:** Comparison of HA titer of influenza and baculoviruses with hemadsorption to insect and mammalian cells expressing HA.

	Hemagglutination titer				Hemadsorption (A540)^1^			
	Influenza virus		Recombinant baculovirus^2^		MDCK cells		Sf9-cells	

	Human	Chicken	Human	Chicken	Human	Chicken	Human	Chicken

OK/323/03	64	0	512	0	0.73 ± 0.07	0.03 ± 0.005	0.71 ± 0.02	0.26 ± 0.06
OK/369/05 wt	64	0	128	0	0.76 ± 0.02	0.20 ± 0.005	0.80 ± 0.09	0.26 ± 0.03
OK/369/05 S138A	N/A^3^	N/A	64	0	N/A	N/A	0.82 ± 0.07	0.31 ± 0.08
OK/372/05	64	32	64	0	0.74 ± 0.004	0.40 ± 0.01	1.05 ± 0.09	0.35 ± 0.06

^1 ^Mean of 4 experiments ± standard deviation

^2 ^Baculovirus was concentrated 10-fold before HA titration

^3 ^N/A = not applicable

### Red cell binding of influenza infected MDCK cells

The difference in chicken red cell binding to HA on Sf9 cells compared to influenza virus might be a function of the lower complexity of N-linked glycans synthesized in insect cells or due to differing density of HA molecules between virus and cell surface. To distinguish between these possibilities, we studied hemadsorption to influenza infected MDCK cells. We found that MDCK cells infected with OK/369/05 wild-type and OK/372/05 bind to chicken red cells, while OK/323/03 infected MDCK cells showed no significant binding to chicken cells (Fig. 4).

The results are summarized in Table 3. The efficiency of binding chicken cells is in the order: HA on Sf9 cells > HA on MDCK cells > influenza virus > HA on baculovirus

### Binding, internalization, and replication of Memphis/31/98 (HG)

We wanted to determine if the ability to agglutinate chicken red cells correlates with ability to infect different cell lines. For these experiments a relatively high concentration of virus is required, so the viruses were grown in chicken eggs. We used Memphis/31/98 (HG), a high growth reassortant with A/NWS/33 internal genes that grows to high titer in chicken eggs yet does not agglutinate chicken red cells, and we compared it to the NWS_HA_-Mem/31/98_NA _(H1N2) reassortant. We do not have Glycoarray results for Memphis/31/98 (HG) but used biotinylated PAA-oligosaccharides to investigate binding specificity as described for NWS-Memphis/31/98 [11].

**Figure 5:**
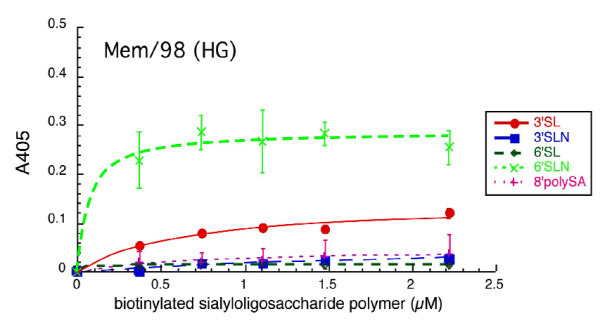
**Binding of biotinylated α2-3- and α2-6- linked sialic acid PAA-compounds to Memphis/31/98 (HG)**. Oligosaccharide bound to virus immobilized in wells of a microtiter plate was detected by AP-streptavidin as described in the Methods. The data points are mean of triplicates ± standard deviation. The line is the fit of the data to the standard binding equation generated using Kaleidagraph software. A similar experiment that shows the binding specificity of NWS-Mem/98 has been published [11].

The results (Fig. 5) show that Memphis/31/98 (HG) binds to 6'-SLN with high affinity (K_d(app) _= 0.14 arbitrary units) and to 3'-SL with low affinity (K_d(app) _= 0.68). Binding to 6'-SL, 3'-SLN, and 8'-polySA is very low. Results for NWS-Mem/98 were reported previously and showed the same specificity except for low affinity binding to 8'-polySA [11].

Binding of virus, internalization, and synthesis of viral protein were studied using a polyclonal antibody that binds nucleoprotein (NP) [37] and Alexa488 labeled secondary antibody, with hemadsorption to detect newly synthesized HA that has been transported to the cell surface. Lectin binding (not shown) indicated that MDCK, HeLa, and A549 cells have both α2-3- and α2-6- sialic acids at the surface but CHO cells stained only with Maackia lectin that binds α2-3-sialic acids in accord with the observation that CHO cells have no 6'sialyltransferase [38].

**Figure 6:**
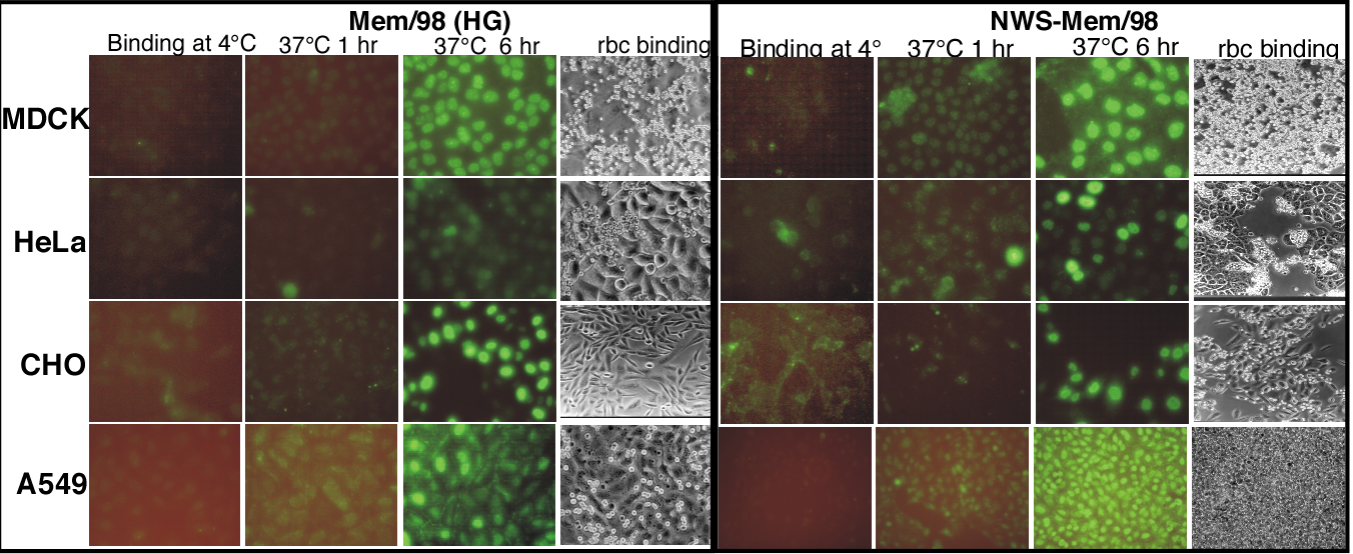
**Binding, internalization and synthesis of viral proteins in different cell lines**. Surface binding of virus (4°C, 1hr), internalization (37°C,1hr), replication to amplify NP (37°C, 6 hr), and cell surface expression of HA seen by hemadsorption at 18 hr (rbc binding). Immuno staining and red cell binding was done as described in Methods.

The results (Fig. 6) show that both Memphis/31/98 (HG) and NWS-Mem/98 viruses can infect all four cell lines, although with different efficiencies. NP was amplified in all cell lines and HA was expressed on the cell surface. Only MDCK cells release measurable virus, as assayed by infectivity.

## Discussion

### Receptor specificity and amino acid changes in the HA1 of recent H3 viruses

A switch in receptor specificity from α2-3- linked sialic acids to α2-6-linked sialic acids has been suggested as necessary for influenza A viruses to adapt from avian to human hosts [39-41]. Mutation of 2 amino acid residues of H3 HA1, Q226L and G228S, was shown to change α2-3 to α2-6 specificity of H3 HA. In H1 HA, "avian" residues Gln226 and Gly228 are present in human viruses, but one amino acid change, E190D of HA1 subunit has been shown to be important in binding the α2-6 human receptor [4,12,19,21,26]. However, H5N1 viruses isolated from humans display the α2-3 receptor-binding properties that are typical of avian viruses [14,15]. This indicates that an avian virus does not need to change receptor specificity to replicate efficiently in humans. The mutations that can convert avian H1 and H3 serotypes to human receptor specificity did not show this switch when they were inserted into H5 HA, although binding to a branched α2-6-biantennary glycan was acquired [15].

Human or avian host specificity was reported to correlate with species of red blood cells bound [21] but in fact chicken red cells were routinely used to measure human virus until the 1990s, when human isolates of H3N2 and H1N1 were found not to agglutinate chicken red cells [18,19,42]. For H3N2 viruses, mutagenesis and hemadsorption studies of A/Aichi/51/92 and A/Chiang Mai/156/88 indicated that a change of E190D of Aichi/92 was responsible for non-agglutination activity with chicken rbcs [19]. Phenotypic variants of A/Paris/97 (H3N2) that reacquired chicken agglutination ability had V226I, and/or S193R and L194I changes [18].

We looked for sequence changes that might be involved in the ability of 2003–2006 H3N2 viruses to agglutinate chicken red cells. Amino acids previously reported to be involved in the loss of chicken red cell agglutination, at positions 190, 193, 194, and/or 226, show no correlation with the red cell binding property. A new N glycosylation site at 144 is present in HA1 of viruses from 2002–2006, but the extra glycosylation is present in all isolates, whether or not they agglutinate chicken red cells. We identified a change of Ala138 to Ser or Thr that was unique to the two 2005 isolates that did not bind chicken red cells, but the mutation S138A in A/OK/369/05 had no effect on the binding properties of the HA.

### Glycan binding analysis

We used fluorescently labeled influenza viruses to probe the Glycan Array to obtain comparative data beyond the α2-3- and α2-6- linkage for these viruses. We expected to find a difference in the glycan binding pattern between chicken red cell agglutinating and non-agglutinating groups, but all the recent viruses in our study, OK/323/03, OK/369/05, OK/370/05, and OK/1992/05, bound to the same group of α2-6-linked oligosaccharides. There was no binding to α2-3-glycans (Fig. 2, Table 1). All the recent human H3N2 viruses, including egg grown A/Wyoming/03/03 X-147, bind to a minimum binding motif: Neu5Acα2-6Galβ1-4GlcNAc, as in polylactosamine structures found on N- and O- linked carbohydrates or Neu5Acα2-6GalNAcβ1-4GlcNAc- (Fig 3A). In general the recent viruses showed higher binding to longer and fucosylated oligosaccharides. The vaccine strain A/Wyoming/03/03 X-147 bound to a somewhat restricted set of α2-6 sialic acids, with low binding to some α2-3 sialylated glycans (Fig 2). The egg-adapted A/Philippines/2/82 X-79 bound to the same set of α2-6 sialylated oligosaccharides as the recent viruses and, in addition, one α2-3 sialylated oligosaccharide with low binding to several other α2-3 structures, a branched α2-6, two biantennary sialylsaccharides branched through mannose at position 4 and two oligosaccharides containing Neu5Gcα2-6Galβ1-4GlcNAc. (Table 2). The binding to Neu5Gc was previously noted to be associated with a mutation of Thr to Tyr at 155 of HA1 [36]. The Tyr155 changed to His in 1989 and then back to Thr in 2002. A/Moscow/99 [34] and the recent viruses studied here no longer bind Neu5Gc.

### Hemagglutination is not the same as hemadsorption

Hemagglutination and hemadsorption generally give the same results [18,43], but we found significant differences in these assays. Table 3 shows that the same HA can bind chicken red cells in one condition (HA expressed on Sf9 cells and/or MDCK cells) and not others (HA on MDCK cells or virus titrated for HA activity). The released baculoviruses that contain the same HA did not agglutinate chicken red cells. It is likely that only a relatively low amount of HA becomes packaged in released baculoviruses, but good titers were obtained with human red cells. Therefore the density of HA may be a critical factor in the ability of a given HA to agglutinate chicken red cells, perhaps determined by other genes since the HA sequences did not show consistent differences.

The interaction between an HA trimer and a monovalent sialylsaccharide is of low affinity (~1 mM) and a large increase in avidity of influenza virus to host cells through multivalent binding via a high density of HA molecules on the virus surface has been assumed [44,45]. Our results suggest that the HA density is higher on the cell surface than on virus particles, perhaps due to the increased curvature of the virion surface. Lectin staining of red blood cells has suggested α2-6 receptors are more abundant on human than chicken red cells and so allow binding even when the HA is less densely packed [19].

### Hemadsorption on insect cells is not the same as on MDCK cells

The lack of chicken red cell hemadsorption activity of OK/323/03 HA expressed on MDCK cell surface, when compared to HA on Sf-9 cells, could potentially be due to the differences in the glycosylation machinery. The smaller glycans on HA expressed in insect cells may allow better access of sialylated receptor molecules to the binding site. Loss of a glycan from HA has been shown to increase its affinity for receptors [46-48]. However, neither the smaller glycans in insect cells nor the action of the viral NA can explain the results in Table 3, leading us to conclude that the change is in avidity rather than affinity and that HA density accounts for the differences.

### Receptor availability does not necessarily lead to productive infection

MDCK, HeLa, and A549 cells have both α2-3- and α2-6- sialic acids at the surface. CHO cells have only α2-3-sialic acids in accord with the observation there is no 6'sialyltransferase [38]. Mem/31/98 and NWS-Mem/98 bind to α2-6-siayllactosamine, but show no or little binding, respectively, to α2-3- sialyllactosamine. Both viruses show some binding to 3'sialyllactose, but this oligosaccharide is not present on cell surface. We expected these viruses to show little or no entry into CHO cells, but Fig 6 shows internalization and production of viral proteins of Mem/31/98 and NWS-Mem/98 in all four cell lines, including CHO cells. There is no release of progeny virus from HeLa, CHO or A549 cells. Productive infection of A/WSN/33 in HeLa cells has been found to be impeded at several stages of replication, including budding and release [49] as we see with Mem/98 and NWS-Mem/98 viruses.

The synthesis of viral proteins of Mem/31/98 in CHO cells shows that α2-6-sialic acid receptors are not necessary for virus infection and replication in the cell, in accord with previous studies that influenza viruses can enter cells in the absence of any surface sialic acid [50]. Cells that have high levels of α2-6 sialic acid on the surface (A549, HeLa) are more efficiently infected but are not permissive for productive infection.

## Conclusion

Our data show that the change to chicken red cell agglutination ability of recent human clinical H3N2 isolates is not associated with a change in receptor specificity to α2-3-sialic acid. We see no consistent changes in HA sequence associated with the binding change and so the change in avidity that led to seemingly retrograde binding to chicken red cells must be subtle; there may be no difference in affinity but the density of HA on Sf9 cells may give significantly increased avidity. This emphasizes the point that receptor binding properties of H3N2 viruses are constantly changing. Our results further show that the presence of high levels of α2-6 receptors on HeLa and A549 cells does not guarantee the production of progeny virus. We conclude that the sialic acid linkage on a cell surface is not the most important determinant of infection.

## Methods

### Viruses

The viruses used in this study are A/OK/323/03 and A/OK/Tf/03 [51], A/OK/369/05, A/OK/370/05, A/OK/371/05, A/OK/372/05, A/OK/1992/05, A/OK/51/05, A/OK/309/05, A/OK/1050/05, A/OK/1472/05, A/Memphis/31/98 (HG) [52], NWS-Mem/31/98 [11]. The recent human viruses were provided to us by Dr. Joseph Waner, Department of Pediatrics, University of Oklahoma Health Sciences Center, Oklahoma City, OK. The viruses were isolated in primary rhesus monkey kidney cells (two passages) and were grown in Madin-Darby canine kidney (MDCK) cells [53]. For comparison we used vaccine strains A/Philippines/2/82 X-79 and A/Wyoming/03/03 X-147 (CDC) grown in embryonated chicken eggs. The viruses were purified by sucrose gradient centrifugation [54] and the purity was checked by SDS gel electrophoresis and silver stain.

### Hemagglutination assay

The MDCK grown viruses were serially diluted in 50 μl of PBS and 50 μl of washed chicken (0.5%) or human (0.8%) red blood cells added. The plates were kept at 4°C and hemagglutination was read at 90 min with human blood and 60 min with chicken blood [55].

### Sequencing

The HA and NA genes of the Oklahoma isolates were amplified by RT-PCR using primers specific for H3 HA and N2 NA. The PCR amplified DNA was gel purified and sequenced at the Oklahoma Medical Research Foundation sequencing facility using primers spaced approximately 500 nucleotides apart. The HA and NA gene sequences of all the viruses have been submitted to the Los Alamos Influenza Sequence Database [56].

### Alexa Fluor-488 labeling of viruses

The purified virus was pelleted out of sucrose and resuspended in CMS (0.15 M NaCl, 0.25 mM CaCl2, 0.8 mM MgCl2) to an HA titer of about 1.0 × 10^5 ^HAU/ml. To 100 μl (~1.0 × 10^4 ^HA units) virus we added 10 μl of 1.0 M sodium bicarbonate pH 9.0. Alexa Fluor-488 succinimidyl ester (Molecular Probes) was added in a ratio of 0.005 μg Alexa per HAU, determined by HA titration to give maximal labeling without loss of binding activity. After stirring for 1 hr at room temperature in the dark, the sample was dialyzed (Slide-A-Lyzer Mini Dialysis Units 7000 MWCO, Pierce) in CaMgS (0.25 mM CaCl_2_, 0.8 mM MgCl_2 _in borate buffered saline, pH 7.2) at 4°C overnight. An aliquot of the dialyzed virus was run on a 9% SDS-PAGE gel to confirm fluorescence of HA1 with no labeling of internal proteins. The HA activity of labeled virus was checked again to make sure binding sites were not inactivated by the reagent.

### Glycan binding of labeled virus

The protocol followed for binding studies is "cfgPTC_197: Printed Glycoarray Screening" [35]. The slide contained a total of 264 glycans: sialylsaccharides, other carbohydrates, and glycoproteins. 50 μl of Alexa-labeled virus was applied to the printed surface of a slide and incubated at 4°C for 1 h. The slide was washed and dried under a stream of nitrogen. The binding image was read in a Perkin-Elmer Microarray XL4000 scanner and analyzed using Imagene (V.6) image analysis software.

### Hemagglutinin cDNA cloning in baculovirus and protein expression

We used the "Bac-to-Bac" Baculovirus Expression System (Invitrogen Life Technologies) to clone the HA gene. The full length HA genes of OK/369/05, OK/323/03, and OK/372/05 were amplified using HA specific forward and reverse primers. The forward primer, 5'-ATAGGCCTGCGGCCGCATGAAGACTATCATTGCTTTG-3' has Stu1 and Not1 restriction enzyme linker at the 5' end and the reverse primer, 5'-GAACTGCAGCAATGGTGATGGTGATGATGAATGCAAATGTTGCACC-3' has a 6-His tag before the stop codon and a Pst1 linker at the 3' end. The PCR amplified HA gene digested with Stu1 and Pst1 then purified by QIAquick PCR Purification Kit (Invitrogen), cloned into the pFastBac vector, and propagated in E. coli (One Shot TOP10; Invitrogen). The Stratagene's QuikChange XL Site-Directed Mutagenesis Kit was used to make OK/369- S138A in the pFastBac plasmid. The recombinant pFastBac plasmids were transformed into DH10Bac™ E. coli cells that contain a baculovirus shuttle vector and a helper plasmid, according to the manufacturer's instructions. The recombinant bacmid DNA was purified from DH10Bac™ cells and used to transfect Sf-9 cells. Expression and functional activity of HA was assayed by red cell binding to BacHA-expressing Sf-9 cells and by hemagglutination titration of baculoviruses.

### Hemadsorption and red cell binding

Sf-9 cells at 80% confluency (in T-25 tissue culture flask) were infected with recombinant HA-baculoviruses. After 5 days of infection they were assayed for red cell binding to surface-expressed HA. The cells were washed 3 times with ice cold CaMgS, fixed lightly with 3% paraformaldehyde for 5 min at 4°C, then washed 3 times with cold PBS. For red cell binding, 4.0 ml of 1% red cells in PBS was added to each flask and incubated for 2 hr at 4°C. The cells were washed several times with PBS until all unbound red cells were removed. To quantitate the amount of red cells bound to each flask, 4.0 ml water was added to the washed cells. After 2 hrs, the cells and debris were spun out at 1,200 g and the clear supernatant was read at 540 nm. Red cell binding to influenza infected MDCK cells in 6 well plates was measured after 18 hrs of infection. To normalize the results to the number of cells, we determined the total soluble protein in Bac-HA infected Sf-9 cells and influenza infected MDCK cells lysed with water. The protein was assayed in cell lysates by Bradford BioRad Protein Assay (BioRad, # 500-0006) and was 0.20 ± 0.004 mg/cm^2 ^for Sf-9 cells and 0.08 ± 0.002 mg/cm^2^for MDCK cells.

### Virus internalization and replication

Cells were grown on cover slips in 35 mm plates. The cells were washed three times with CaMgPBS and infected with 300 μl freshly grown virus from MDCK cells. The cells were incubated at 4° for 1 hr to allow the virus to bind to the cell surface and then transferred to 37°C for 1 hr, 6 hr, and 18 hr. After 1 hr the virus is internalized and NP can be detected as it concentrates in the nucleus. By 6 hr post-infection new NP has been synthesized and higher levels are seen in the nucleus. By 18 hr there is a sufficient surface expression of HA for red cells to bind. To detect NP the cells were washed twice with cold PBS and fixed with 1 ml freshly prepared 3% paraformaldehyde in PBS, on ice for 5 min. After washing three times with cold PBS, cells were permeabilized with 2% triton X-100 in PBS for 10 min at room temperature, washed 3 times with cold PBS, once with 50 mM ammonium chloride in PBS to destroy any remaining paraformaldehyde, and blocked with 10% supplemented calf serum in PBS for 15 min at room temperature. Fifty μl of rabbit anti-core (mostly anti-NP) antiserum diluted 1:1000 in 0.1% BSA/PBS was added for 30 min at RT. After washing with 0.1% BSA in PBS, cells were incubated with 50 μl Alexa Fluor-488 labeled goat anti-rabbit antiserum (1:200 dilution) for 30 min at room temperature. After washing five times with 0.1% BSA/PBS and once with water, the cover slip was drained well and mounted with a small drop of Vectashield (Vector Laboratories Burlingame, CA). The slides were examined and recorded on a Fluorescent Diaphot-300 (Nikon Corporation) microscope with a Nikon Dxm1200 digital camera.

### Binding of Memphis/31/98 (HG) and NWS-Mem/31/98 to biotinylated PAA-oligosaccharides

Binding was measured as previously described [11]. The ELISA plate was coated with 32 HAU of purified virus in 50 μl 0.15 M sodium carbonate, 0.3 M sodium bicarbonate, pH 9, and incubated at 4°C overnight. The plate was washed with PBS, blocked with 1% BSA in PBS for 1 hr, and washed with PBS. Fifty μl of various dilutions of biotinylated PAA oligosaccharide was added to give the desired final concentration. After 1 hr incubation on ice the plate was washed and the bound oligosaccharide was quantitated by alkaline phosphatase-conjugated streptavidin. The biotinylated oligosaccharides used were:

Neu5Acα2-3Galβ 1,4Glc (3'SL, 3' sialyllactose); Neu5Acα2-3Galβ 1,4GlcNAc (3'SLN, 3' sialyllactosamine); Neu5Acα2-6Galβ 1,4Glc (6'SL, 6' sialyllactose); Neu5Acα2-6Galβ1,4GlcNAc (6'SLN, 6' sialyllactosamine); Neu5Acα 2,8Neu5Acα 2,8Neu5Acα (8'polySA, trisialic acid).

## Competing interests

The author(s) declare that they have no competing interests.

## Authors' contributions

KK carried out the red cell binding, mutagenesis and baculovirus expression studies, and drafted the manuscript. SG carried out the internalization studies and HA titrations, UG helped with labeling of virus. DFS and RDC designed and supervised the Glycan Array studies and data analyses. GMA conceived the study, designed several of the experiments and completed the manuscript.
